# Reduced telomere length is associated with fibrotic joint disease suggesting that impaired telomere repair contributes to joint fibrosis

**DOI:** 10.1371/journal.pone.0190120

**Published:** 2018-01-02

**Authors:** Nicholas S. Kalson, Tim M. Brock, Massimo Mangino, Stella M. Fabiane, Derek A. Mann, Lee A. Borthwick, David J. Deehan, Frances M. K. Williams

**Affiliations:** 1 Fibrosis Research Group, Institute of Cellular Medicine, University of Newcastle, Newcastle upon Tyne, United Kingdom; 2 Department of Orthopaedics, Newcastle University Hospitals NHS Foundation Trust, Newcastle upon Tyne, United Kingdom; 3 Department of Twin Research & Genetic Epidemiology, King’s College London, London, United Kingdom; 4 NIHR Biomedical Research Centre at Guy’s and St Thomas’ Foundation Trust, London, United Kingdom; Centre National de la Recherche Scientifique, FRANCE

## Abstract

**Objective:**

Joint fibrosis affects many synovial joints (including hip, knee and shoulder) causing stiffness and pain. The mechanism of joint fibrosis remains unknown, although genetic factors may contribute. Defects in maintenance of telomere length resulting from impaired telomere repair have been shown to cause lung and liver fibrotic disease. Here we tested the hypothesis that joint fibrosis and other soft tissue fibrotic conditions are also associated with telomere length.

**Patients and methods:**

5,200 participants in the TwinsUK registry had data on telomere length (measured by qPCR) and the traits of interest (hip and knee stiffness, total joint replacement (TJR, hip or knee) and fibrotic conditions (Dupuytren’s disease, frozen shoulder).

**Results:**

Multivariable logistic regression analyses showed a significant association between telomere length and fibrotic conditions (hip stiffness, knee stiffness and frozen shoulder, p = ≤0.002) even after taking age into account. No association was found between TJR and telomere length.

**Conclusion:**

These findings suggest that defects in telomere repair contribute to joint fibrosis, and that fibrosis shares a common mechanistic pathway in different organs. Therapeutic strategies to combat telomere shortening may offer novel treatments for fibrotic joint disease.

## Introduction

Using the TwinsUK registry of monozygotic and dizygotic twins [1], we have recently found a significant association between fibrotic soft tissue conditions, joint stiffness and total joint arthroplasty [2]. Fibrotic soft tissue conditions (frozen shoulder, Dupuytren’s disease, reported hip and knee joint stiffness) appear to be polygenic disease traits with a heritability of ~40% [2]. However, the molecular mechanisms driving joint fibrosis remain unknown and therapeutic options are extremely limited [3,4].

The process of fibrosis is characterized by the deposition of a dense, disorganized extracellular matrix of collagen [5]. In fibrotic conditions affecting the joints, such as adhesive capsulitis [6] and joint fibrosis [7], this manifests itself as joint stiffness, which is hugely debilitating and adversely affects quality of life. Whilst the aetiology is multifactorial, it is likely that different genetic and environmental triggers converge on a ‘common pathway’, involving α-smooth muscle actin containing myofibroblasts, TGF-1β signaling and rapid deposition and tensioning of the new matrix [8]. To date, treatment strategies have largely focused on debridement and/ or distension of the extracellular matrix. However, the underlying pathogenic mechanisms of disease are not understood and thus targeted treatment has not yet been developed [3,4].

Recent research suggests that defects in telomere maintenance involving the telomerase reverse transcriptase (TERT) gene cause liver and lung fibrotic disease [9]. It is unclear at present whether the joint soft tissue fibrosis is also telomere-mediated. Using telomere length and existing information in the TwinsUK database we tested the hypothesis that soft tissue fibrosis shares common telomere related aetiology as telomere-related lung and liver fibrosis.

## Patients and methods

Participants were selected from the TwinsUK registry [10] on the availability of data from four different questionnaires and clinical visits between 1992 and 2008. TwinsUK is the UK's largest registry of monozygotic (MZ) and dizygotic (DZ) twins. It contains extensive genotype and phenotype data obtained at clinical visits and by mailed and online questionnaires. The twin characteristics have been shown to be similar to the general singleton population for a range of traits and lifestyle factors [10]. Responses to the questions ‘Have you ever had pain or stiffness in the following joints? left knee/right knee/left hip/right hip’; ‘have you undergone a total knee or total hip replacement?’; and ‘been diagnosed with frozen shoulder or Dupuytren’s contracture?’ (fibrotic conditions) were extracted. Age, sex and body mass index (BMI) were also extracted for the time-point relevant to each questionnaire. Non-respondents for each particular condition were considered not to have the condition, and were coded as negatives. This standard data-handling practice in the TwinsUK dataset precludes descriptive analysis of ‘non-responders’. Participants were not aware of a specific hypothesis related to joint stiffness or joint replacement being tested in this study, nor was the temporal relationship of the traits explored. Ethics committee approval for the study was obtained from St Thomas’ Hospital Ethical Review Board. All participants gave written, informed consent. King’s College Hospital approved the consent procedure.

### Telomere length

Mean leukocyte telomere length (LTL) was measured using a qPCR-based technique as part of a larger study using the TwinsUK cohort as previously described [11]. Full details of the method are included in Supplementary Information 1 (S1 Method). This method expresses telomere length as a ratio (T/S) of telomere repeat length (T) to copy number of a single copy gene (S), in each sample. To standardise across plates, either a calibrator sample or a standard curve was used for quantification. Mean LTL was first assessed for age-related shortening and for an association of longer LTL with female sex in all cohorts, and showed expected associations. LTLs were standardised using a Z-score transformation and adjusted for age and sex. To reduce the site-dependent effect one telomere length all measurements in this cohort were made in one specialised centre (Leicester) as part of a large (> 30,000 subject) meta-analysis study [11].

### Statistical analysis

Logistic regression analysis was used to determine the association between the three traits of interest (joint stiffness, TJR and fibrotic conditions) and telomere length. Adjustment was made for age, sex, BMI and smoking status. The cluster option in Stata was used to adjust for family relatedness (as previously described [12–14]. This model provides a robust estimation of the standard errors taking into account that the observations within families are not independent. For the purposes of this analysis, missing data was assumed to be negative. Statistical analysis was performed using Stata software (StataCorp, Texas, USA).

## Results

The soft tissue sample comprised 9,718 twins with information on at least one trait at any time-point (hip or knee stiffness, fibrotic condition, TJR) (Table 1). This included 287 with a stiff joint, 762 with a fibrotic condition, 378 with TJR. The mean age of the sample was 47.2 years and 87.8% were female. Measurement of TL was available for a total of 5634 twins having soft tissue information (58%).

**Table 1:** Demographic characteristics of the TwinsUK sample.

	Total
**Total respondents**	5989
Mean age	49.0 (16.5–99)
Mean BMI	25.4 (sd 4.7)
No of females	5422 (90.5%)
Stiff knee	185
Stiff hip	116
Stiff joint (total)	239
Frozen shoulder	549
Dupuytren's contracture	19
Fibrotic condition	559
TKR	144
THR	258
TJR	300

There were no significant differences in age, BMI or sex between the zygosity groups. TKR represents total knee replacement; THR, hip replacement; TJR, total joint (either knee or hip) replacement.

### Univariable analysis

Logistic regression analysis was performed with telomere length as the predictor variable (Table 2), for each of the 7 phenotypes. Respondents reporting hip stiffness or knee stiffness had a significant association with TL (OR 0.68, 95% CI 0.58–0.78 and OR 0.73, 95% CI 0.59–0.89, p <0.001 and 0.002 respectively). Respondents with frozen shoulder had a significant relationship with TL (OR 0.78 CI 0.70–0.85, p = <0.001). No association was detected between Dupuytren’s disease and TL (OR 0.90, CI 0.65–1.25, p = 0.532). Presence of TJR (hip and knee) did not show an association with TL (OR 0.95, CI 0.83–1.08, p = 0.42).

**Table 2:** Results of logistic regression analyses with telomere length as the predictor, in 5634 twins.

	Odds ratio	Standard Error	z-score	p -value	95% confidence intervals
Knee stiffness	0.680	0.543	-4.83	0.000	0.581–0.795
Hip stiffness	0.728	0.076	-3.06.	0.002	0.594–0.892
Dupuytren’s	0.900	0.152	-0.63	0.532	0.645–1.254
Frozen shoulder	0.775	0.038	-5.20	0.000	0.703–0.853
TKRs	0.897	0.082	-1.18	0.237	0.749–1.074
THR	0.967	0.783	-0.41	0.682	0.826–1.082
TJR	0.947	0.064	-0.80	0.423	0.829–1.082

All analyses were age and sex and BMI adjusted, as well as taking familial relationship into account.

## Discussion

Here we report a significant association between telomere length and the presence of fibrotic joint disease in a large sample taken from the NIHR BRC TwinsUK BioResource. This suggests that impaired telomere maintenance may contribute to the development of joint fibrosis. Currently our understanding of the pathogenesis of joint fibrosis is poorly understood and consequently treatment options are severely limited. Measurement of TL by qPCR is a robust method and these twin TL data have previously contributed to the identification of new genetic loci associated with reduced TL in a genome-wide association study[15]. In addition TwinsUK were one of seven independent samples to demonstrate to demonstrate a relationship between TL and leptin levels [16].

Analysis of this observational study has limitations. Firstly, diagnosis of fibrotic joint disease relied on patient-reported data. However, the incidence of these conditions was found to be similar to previous studies [6,17]. Secondly, missing data was assumed to be negative, which may have underestimated incidence values. This biases the dataset towards the null, making positive findings less, rather than more, likely. Finally, while the overall sample size was generous, there were few cases of Dupuytren’s compared to the other conditions, which may explain the non-significant findings for this phenotype.

Other fibrotic conditions such as liver cirrhosis and pulmonary fibrosis have been shown to have a genetic disposition, with mutations seen in the TERT gene responsible for telomere maintenance and repair [18]. As a result, these patients have short telomeres, with a quantitative defect in stem-cell number and a qualitative deficiency in stem-cell regeneration [9]. Sex hormones have been shown to regulate the expression of the telomerase gene in tissue [19] and animal models [20], and more recently, resulted in telomere elongation at 24 months in a phase 1–2 trial [9]. Soft tissue fibrotic conditions have not previously been considered part of this spectrum, although recent evidence suggests they are heritable [2]. It is plausible that they share underlying genetic variants, and this work suggests that telomere shortening genetic variants should be explored. Our work also provides a rationale for testing the new therapeutic agents that attenuate telomere shortening in patients with joint fibrosing conditions.

## Supporting information

S1 Method qPCR telomere length measurement. A detailed description of the qPCR method used to measure telomere length is provided together with relevant references.
(DOCX)
