# Supplementary material for: Reduced telomere length is associated with fibrotic joint disease suggesting that impaired telomere repair contributes to joint fibrosis
Source: PLoS One. 2018 Jan 2;13(1):e0190120. doi: 10.1371/journal.pone.0190120 (PMC5749754; doi:10.1371/journal.pone.0190120)
Supplement: S1 Method — A detailed description of the qPCR method used to measure telomere length is provided together with relevant references. (DOCX) [file pone.0190120.s001.docx]

S1 Method. qPCR Telomere Length Measurement

Mean leucocyte telomere length (LTL) was measured using an established and validated quantitative PCR-based technique [1,2]. All LTL measurements were performed as part of a larger study using the TwinsUK cohort in which more than 32,000 individual’s samples were analysed[1]. qPCR telomere length measurements from this sample have been reported previously [1,3-5].

Telomere length is expressed as a ratio (T/S) of telomere repeat length (T) to copy number of a single copy gene, 36B4(S), within each sample. DNA samples were run in duplicate in 25μL reactions containing 1x SensiMix NoRef SYBR Green master mix (Quantace, UK), 600nM of Tel1b, 600nM Tel2b primers and 30ng of template DNA. For the 36b4 PCR the above was performed, substituting the Tel primers for 300nM 36b4U and 500nM of 36B4D (for details of primer sequences please see [5]).

Reactions were set up using a CAS-1200 liquid handling system and run on a Rotorgene 6000 Real Time Thermal Cycler (both Qiagen, UK). Alongside the samples each run also contained a calibrator sample (K562 cell line DNA) in duplicate and a no template control. Cycling conditions were 95**°**C incubation for 10mins followed by either 20 cycles (telomere) or 30 cycles (36B4) of 95**°**C for 15 sec and 58**°**C for 1min. Dilution series (100-1.56ng in two-fold dilutions) were run for both telomere and 36B4 assays to establish the linear range. Good linearity was observed across this range (R^2^ > 0.99) and the input amount of 30ng was subsequently set. Any samples found to run outside this range were diluted and run again.

Analysis of the PCR output was performed using Comparative Quantitation (Qiagen Rotorgene analysis software, Qiagen, UK) as previously described [1,5]. Briefly, a takeoff value was produced for each sample based on the second derivative of the amplification plot (the start of the exponential phase). The amplification efficiency for each sample and subsequently the mean amplification across the run was calculated. The relative concentration of the sample was then calculated relative to the calibrator sample using the takeoff value and amplification efficiency. This was performed for both the telomere (T) and 36B4 (S) assays and telomere length expressed as the ratio of these (T/S). T/S distribution was 3.71±0.68 (mean+/- SD), range was 0.68 – 11.40[1].

For quality control all samples were checked for concordance between duplicate values. Samples showing a difference between the duplicate measurements of greater than 0.1 cycles in the takeoff value were excluded and re-run. The coefficient of variation between duplicate measurements was 1.9% (T), 1.6% (S) and 2.9% (T/S ratio). In addition, to test reproducibility of the assay, multiple samples were randomly chosen to be run again on a separate day and checked for agreement between the two runs. Reproducibility data gave good agreement between the T/S ratios from the two (R2 = 0.90, P<0.0001) and gave an inter-run coefficient of variation of between 2.7% and 3.9%%[1]

1. Codd V, Nelson CP, Albrecht E, Mangino M, Deelen J, Buxton JL, et al. Identification of seven loci affecting mean telomere length and their association with disease. Nat Genet. Nature Research; 2013;45: 422–7– 427e1–2. doi:10.1038/ng.2528

2. Cawthon RM. Telomere measurement by quantitative PCR. Nucleic Acids Res. 2002;30: e47.

3. Mangino M, Christiansen L, Stone R, Hunt SC, Horvath K, Eisenberg DTA, et al. DCAF4, a novel gene associated with leucocyte telomere length. J Med Genet. BMJ Publishing Group Ltd; 2015;52: 157–162. doi:10.1136/jmedgenet-2014-102681

4. Albrecht E, Sillanpää E, Karrasch S, Alves AC, Codd V, Hovatta I, et al. Telomere length in circulating leukocytes is associated with lung function and disease. Eur Respir J. European Respiratory Society; 2014;43: 983–992. doi:10.1183/09031936.00046213

5. Codd V, Mangino M, van der Harst P, Braund PS, Kaiser M, Beveridge AJ, et al. Common variants near TERC are associated with mean telomere length. Nat Genet. Nature Research; 2010;42: 197–199. doi:10.1038/ng.532
